# Time course of lactate clearance in trauma and its relevance to outcomes

**DOI:** 10.1186/1757-7241-20-S1-O9

**Published:** 2012-03-22

**Authors:** MS Chana, J Manson, R Davenport, HD De'Ath, C Spoors, I Raza, S Khan, A Coates, C Rourke, K Brohi

**Affiliations:** 1 The Blizard Institute of Cell and Molecular Science, Barts and The London School of Medicine and Dentistry, UK

## Introduction

Serum lactate is raised during haemorrhage of trauma and high admission levels are associated with worse outcomes. The time-course of lactate clearance and the effect of packed red blood cell transfusion (PRBC) has not previously been described. This information is important for clinical trials of novel oxygen therapeutics.

## Methods

A prospective observational cohort study of trauma patients was performed. Blood was drawn pre-resuscitation and after 4, 8 and 12 units of PRBC for serum lactate levels, and at 24 & 72 hours. Patients were stratified based on admission lactate & on the time to normalisation. Clearance profiles and outcomes were compared.

## Results

Of 300 patients enrolled, 50 received 4 or more units of PRBCs. On average, patients with high admission lactates (>=5.0mmol/l) did not clear their lactate until blood transfusion had stopped and haemorrhage was controlled (p <0.05. Figures A and B – patients receiving 4 and 8 units of PRBCs). Patients who normalised their lactate levels prior to the end of transfusion had significantly reduced mortality compared to those not clearing although this did not reach statistical significance due to small numbers (mortality for patients receiving PRBC cleared vs not cleared: PRBC 4u: 33% vs 43%; PRBC 8u: 50% vs 63%; PRBC 12u: 67% vs 71%).

## Conclusions

Lactate levels tend to remain elevated during trauma haemorrhage and PRBC transfusion. Mortality is lower in patients whose lactate normalises during transfusion. Our study provides important information about patient resuscitation and for design of future clinical trials of novel oxygen therapeutics.

